# Leucopenia and treatment efficacy in advanced nasopharyngeal carcinoma

**DOI:** 10.1186/s12885-015-1442-3

**Published:** 2015-05-24

**Authors:** Zhen Su, Yan-Ping Mao, Pu-Yun OuYang, Jie Tang, Xiao-Wen Lan, Fang-Yun Xie

**Affiliations:** 1 Sun Yat-sen University Cancer Center, State Key Laboratory of Oncology in South China, Collaborative Innovation Center for Cancer Medicine, Guangzhou, 510060 China; 2 Department of Radiation Oncology, Sun Yat-sen University Cancer Center; State Key Laboratory of Oncology in South China; Collaborative Innovation Center for Cancer Medicine, Guangzhou, 510060 China

**Keywords:** Leucopenia, Advanced nasopharyngeal carcinoma, Chemoradiotherapy, Survival, Treatment efficacy

## Abstract

**Background:**

Leucopenia or neutropenia during chemotherapy predicts better survival in several cancers. We aimed to assess whether leucopenia could be a biological measure of treatment and a marker of efficacy in advanced nasopharyngeal carcinoma (ANPC).

**Methods:**

We retrospectively analyzed 3826 patients with ANPC who received chemoradiotherapy. Leucopenia was categorised on the basis of worst grade during treatment according to the National Cancer Institute Common Toxicity Criteria version 4.0: no leucopenia (grade 0), mild leucopenia (grade 1–2), and severe leucopenia (grade 3–4). Associations between leucopenia and survival were estimated by Cox proportional hazards model.

**Results:**

Of the 3826 patients, 2511 (65.6 %) developed mild leucopenia (grade 1–2) and 807 (21.1 %) developed severe leucopenia (grade 3–4) during treatment; 508 (13.3 %) did not. A multivariate Cox model that included leucopenia determined that the hazard ratios (HR) of death for patients with mild and severe leucopenia were 0.69 [95 % confidence interval (95 %CI) 0.56-0.85, *p* < 0.001] and 0.75 (95 %CI 0.59-0.95, *p* = 0.019), respectively; the HR of distant metastasis for patients with mild and severe leucopenia were 0.77 (95 %CI 0.61-0.96, *p* = 0.023) and 0.99 (95 %CI 0.77-1.29, *p* = 0.995), respectively. Leucopenia had no effect on locoregional relapse.

**Conclusions:**

Our results indicate that mild leucopenia during chemoradiotherapy is associated with improved overall survival and distant metastasis–free survival in ANPC. Mild leucopenia may indicate appropriate dosage of chemotherapy. We can identify the patients who may benefit from chemotherapy if they experienced leucopenia during the treatment. Prospective trials are required to assess whether dosing adjustments based on leucopenia may improve chemotherapy efficacy.

## Background

Nasopharyngeal carcinoma (NPC) is a distinct type of head and neck cancer. The incidence rate is as high as 20–30 per 100,000 populations in endemic areas of southern China and Southeast Asia [1–3]. Radiotherapy (RT) is the primary treatment, plus chemotherapy when needed according to clinical stage. With the development of diagnostic imaging, chemotherapy regimens, targeted drugs, and radiotherapeutic techniques, especially the application of IMRT (Intensity Modulated Radiation Therapy), survival of NPC has improved significantly [4–6]. However, 10–20 % of patients with advanced NPC (ANPC) develop distant metastasis after radical chemoradiotherapy, rendering distant metastases the main reason for treatment failure. To reduce the occurrence of distant metastasis, different timings of chemotherapy is recommended for ANPC according to NCCN (National Comprehensive Cancer Network) guidelines [7]. In 2014 version of NCCN guidelines , the categories of evidence for induction or adjuvant chemotherapy of NPC has changed [7]. Category of induction chemotherapy of NPC changed from category 2A to category 3. Category of adjuvant chemotherapy “cisplatin + RT followed by cisplatin/5-FU changed from category 1 to category 2A and “cisplatin + RT followed by carboplatin/5-FU changed from category 2A to category 2B.

Bone marrow suppression is a common adverse reaction of cytotoxic drugs and could be a biological measure of drug activity and might predict treatment efficacy [8, 9]. Leucopenia or neutropenia during treatment is a common phenomenon of bone marrow suppression. Some studies reported that leucopenia or neutropenia is a prognostic factor predicting better clinical outcome in several solid tumors, e.g., breast cancer [10–12], colorectal cancer [13, 14], advanced gastric cancer [15–17], lung cancer [18–20], and Hodgkin’s lymphoma [21]. Others have reported different results [22, 23]. However, the predictive (ie, estimation of the chance of benefit from chemotherapy) or prognostic (ie, estimation of the chance of survival) role of leucopenia in advanced nasopharyngeal carcinoma have not been established.

We aimed to investigate the association between leucopenia during treatment and survival of ANPC and to provide evidences, through rigorous statistical analysis of a large series of subjects with ANPC, of the utility of leukocyte count as a surrogate marker of drug efficacy.

## Methods

### Patients and methods

We retrospectively collected 3939 newly diagnosed ANPC patients from January 2005 to December 2010 treated in the Nasopharyngeal Carcinoma Department of Sun Yat-Sen University Cancer Center. 113 paitents were excluded owing to different reasons, abnormal liver function, abnormal kidney function, unsatisfactory blood sugar control and so on. 3826 patients were involved in the study. The Sun Yat-Sen University Cancer Center Institutional Review Board (IRB) and ethics committee reviewed and approved the study. The study was retrospective. Patient records were anonymized and de-identified prior to analysis.

Pretreatment evaluation included complete patient history, physical examination, hematology and biochemistry profiles, nasopharynx and neck magnetic resonance imaging (MRI), chest radiography, abdominal ultrasound, bone emission computed tomography (ECT), and chest or abdomen computed tomography (CT) when necessary.

### Treatment

The treatment strategy for all patients was based on National Comprehensive Cancer Network Guidelines [24, 25]. All patients were treated with intensity-modulated RT (IMRT) or conventional RT (CRT) with chemotherapy; the radiation techniques and chemotherapy regimens have been described previously [26, 27].

### Laboratory measurements

We performed leukocyte and neutrophil counts for all patients within two weeks before therapy and at least once weekly during treatment. The most severe grade of leucopenia was based on the lowest recorded leukocyte count for a given patient between the first day of treatment administration and 1 week after the end of treatment, and was graded according to the National Cancer Institute Common Toxicity Criteria version 4.0. Patients were classified as having no leucopenia (grade 0), mild leucopenia (grade 1–2), and severe leucopenia (grade 3–4). Indications for using granulocyte colony–stimulating factor (G-CSF) were not specified; it was generally used in grade 3–4 or febrile leucopenia, and was not used for prophylaxis.

### Follow-up

Patients were regularly followed after RT until death or their last follow-up appointment. Clinic visits were scheduled every three months in the first three years, every six months during the fourth to fifth years, and once a year after the fifth year. Patients underwent physical examination and nasopharyngoscopy on each visit. Nasopharynx and neck MRI, chest radiography, abdominal ultrasound, and ECT were performed after RT or according to clinical indications. The follow-up duration was calculated from the first day of therapy to the day of death or the day of last examination.

### Statistical analysis

We estimated the following endpoints (interval to the first defining event): overall survival (OS), locoregional relapse–free survival (LRFS), and distant metastasis–free survival (DMFS). Survival curves were estimated using the Kaplan-Meier method and compared using the log-rank test. Multivariate analyses were performed using the Cox proportional hazards model. We used chi-square tests and Kruskal–Wallis H tests to assess the statistical significance of associations between categorical variables and the three groups. All statistical tests were 2-tailed; *p* < 0.05 was considered statistically significant. All tests were conducted using IBM SPSS version 20.0.0 (IBM Corporation, Armonk, NY, USA).

## Results

### Patient characteristics

Table 1 lists the patient characteristics. We studied 3826 patients (2873 male; 953 female). The median age at diagnosis for male patients was 46 years (range 20–84 years); that for female patients was 44 years (range 20–76 years). CRT and IMRT were administered to 2583 and 1243 patients, respectively. Induction chemotherapy (IC) was administered to 1073 patients, concurrent chemotherapy (CC) to 1291 patients, IC plus CC (IC + CC) to 1255 patients, and CC plus adjuvant chemotherapy (CC + AC) to 207 patients. We administered <4 and ≥4 chemotherapy cycles to 2364 (61.8 %) and 1462 (38.2 %) patients, respectively. No significant differences were observed for age, T classification, N classification, and clinical stage. There were significant differences in pretreatment leukocyte count, type of chemotherapy, chemotherapy cycles, type of RT, sex, and paclitaxel use (yes or no) in the compared groups (all *p* < 0.05). Patients who developed leucopenia during treatment had lower pretreatment leukocyte counts (*p* < 0.001). More female patients developed leucopenia (female vs. male, 90.1 % vs. 85.3 %, *p* < 0.001); patients using paclitaxel were likely to develop severe leucopenia (31.4 % vs. 18.4 %, *p* < 0.001).

**Table 1:** Patient characteristics according to grade of leucopenia

Variable	All	Absent leucopenia	Mild leucopenia	Severe leucopenia	*P* value
Total	3826	508(13.3)	2511(65.6)	807(21.1)	
Gender					<0.001
male	2873(75.1)	421(82.9)	1936(77.1)	516(63.9)	
female	953(24.9)	87(17.1)	575(22.9)	291(36.1)	
Age(years)					0.105
<45	1982(51.8)	242(47.6)	1325(52.8)	415(51.4)	
> = 45	1844(48.2)	266(52.4)	1186(47.2)	392(48.6)	
Leukocyte count					<0.001
= < 10 × 10^9/L	3448(90.1)	424(83.5)	2278(90.7)	746(92.4)	
>10 × 10^9/L	378(9.9)	84(16.5)	233(9.3)	61(7.6)	
Pathological type(WHO)					0.692
I	83(2.2)	11(2.2)	55(2.2)	17(2.1)	
II	203(5.3)	23(4.5)	143(5.7)	37(4.6)	
III	3540(92.5)	474(93.3)	2313(92.1)	753(93.3)	
T-classification					0.720
T1	172(4.5)	22(4.3)	109(4.3)	41(5.1)	
T2	272(7.1)	29(5.7)	185(7.4)	58(7.2)	
T3	1871(48.9)	271(53.3)	1221(48.6)	379(47.0)	
T4	1511(39.5)	186(36.6)	996(39.7)	329(40.8)	
N-classification					0.09
N0	517(13.5)	86(16.9)	335(13.3)	96(11.9)	
N1	1978(51.7)	252(49.6)	1313(52.3)	413(51.2)	
N2	1043(27.3)	133(26.2)	680(27.1)	230(28.5)	
N3	288(7.5)	37(7.3)	183(7.3)	68(8.4)	
Clinical stage					0.222
III	2094(54.7)	295(58.1)	1369(54.5)	430(53.3)	
IV	1732(45.3)	213(41.9)	1142(45.5)	377(46.7)	
Radiotherapy					0.004
CRT	2583(67.5)	329(64.8)	1741(69.3)	513(63.6)	
IMRT	1243(32.5)	179(35.2)	770(30.7)	294(36.4)	
Chemotherapy					<0.001
IC	1073(28.0)	198(39.0)	697(27.8)	178(22.1)	
CC	1291(33.7)	202(39.8)	878(35.0)	211(26.1)	
IC + CC	1255(32.8)	98(19.3)	804(32.0)	353(43.7)	
CC + AC	207(5.4)	10(2.0)	132(5.3)	65(8.1)	
Paclitaxel					<0.001
NO	3029(79.2)	403(79.3)	2069(82.4)	557(69.0)	
YES	797(20.8)	105(20.7)	442(17.6)	250(31.0)	
Chemotherapy cycles					<0.001
<4	2364(61.8)	400(78.7)	1575(62.7)	389(48.2)	
> = 4	1462(38.2)	108(21.3)	936(37.3)	418(51.8)	

Abbreviations: CRT: conventional radiotherapy; IMRT: intensity modulated radiation therapy; IC: Induction chemotherapy; CC: concurrent chemotherapy; AC: adjuvant chemotherapy; WHO: world health organization

The median OS was 52.6 months (range 3.07–113.0 months); 10.9 % of patients (417/3826) developed locoregional relapse, 16.5 % (633/3826) developed distant metastases, and 19.0 % (727/3826) died. The 5-year OS, LRFS, and DMFS rates for the entire population were 80.70 %, 87.9 %, and 82.1 %, respectively.

During treatment, 2511 patients (65.6 %) developed mild leucopenia (grade 1–2) and 807 patients (21.1 %) developed severe leucopenia (grade 3–4); the remaining 508 (13.3 %) did not develop leucopenia.

### Survival analyses including leucopenia

**Fig. 1:**
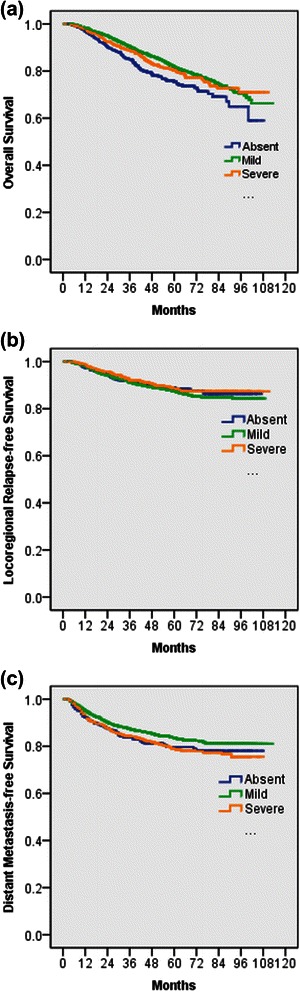
Kaplan–Meier survival curves of (**a**) Overall Survival, (**b**) Locoregional Relapse-free Survival, and (**c**) Distant Metastasis-free Survival according to severity of leucopenia

Table 2 shows the univariate analysis of the baseline and clinical characteristics as prognostic factors, including leucopenia. Kaplan–Meier curves according to severity of leucopenia showed that better OS and DMFS were predicted for patients with leucopenia and that leucopenia had no significant effect on LRFS (Fig. 1). The 5-year OS rate in patients with no leucopenia, mild leucopenia, and severe leucopenia was 75.5 %, 81.9 %, and 80.5 %, respectively (mild vs no leucopenia, *p* = 0.001; severe vs no leucopenia, *p* = 0.03; mild vs severe, *p* = 0.314). The 5-year DMFS rate in patients with no leucopenia, mild leucopenia, and severe leucopenia was 79.7 %, 83.7 %, and 78.9 %, respectively (mild vs. no leucopenia, *p* = 0.038; severe vs no leucopenia, *p* = 0.927; mild vs severe, *p* = 0.007). The 5-year LRFS rate in patients with no leucopenia, mild leucopenia, and severe leucopenia was 88.9 %, 87.4 %, and 88.6 %, respectively (all *p* > 0.05 for any two compared groups).

**Table 2:** Univariate analysis of survival for patients with ANPC

	All population		Cycles <4 population		Cycles > =4 population	
Variable	OS	DMFS	OS	DMFS	OS	DMFS
Leucopenia						
Mild VS Absent						
HR(95 %CI)	0.70(0.57-0.86)	0.79(0.63-0.98)	0.73(0.57-0.92)	0.87(0.66-1.14)	0.56(0.38-0.86)	0.56(0.37-0.86)
*p*	0.001	0.038	0.009	0.309	0.007	0.008
Severe VS Absent						
HR(95 %CI)	0.77(0.60-0.97)	1.01(0.78-1.31)	0.86(0.63-1.16)	1.10(0.79-1.54)	0.59(0.38-0.90)	0.73(0.46-1.14)
*P*	0.030	0.927	0.320	0.554	0.016	0.166
Mild VS Severe						
HR(95%CI)	0.91(0.76-1.09)	0.77(0.64-0.93)	0.85(0.66-1.08)	0.78(0.61-1.02)	0.98(0.75-1.28)	0.77(0.58-1.09)
*p*	0.314	0.007	0.191	0.069	0.887	0.058
Gender						
HR(95 %CI)	0.62(0.51-0.75)	0.69(0.57-0.84)	0.63(0.50-0.79)	0.71(0.55-0.91)	0.61(0.45-0.84)	0.67(0.49-0.2)
*P*	<0.001	<0.001	<0.001	0.007	0.002	0.015
Age						
HR(95 %CI)	1.84(1.59-2.14)	1.09(0.93-1.27)	1.93(1.59-2.34)	1.14(0.94-1.39)	1.73(1.36-2.19)	1.01(0.77-1.30)
*P*	<0.001	0.304	<0.001	0.191	<0.001	0.941
T-classification						
HR(95 %CI)	1.27(1.14-1.40)	1.09(0.99-1.22)	1.27(1.11-1.45)	1.11(0.96-1.27)	1.26(1.07-1.49)	1.08(0.91-1.27)
*P*	<0.001	0.092	0.001	0.157	0.007	0.375
N-classification						
HR(95CI)	1.56(1.43-1.70)	1.65(1.50-1.81)	1.70(1.51-1.90)	1.75(1.54-1.97)	1.39(1.21-1.60)	1.52(1.32-1.76)
*P*	<0.001	<0.001	<0.001	<0.001	<0.001	<0.001
Radiotherapy						
HR(95 %CI)	0.80(0.68-0.94)	0.91(0.76-1.08)	0.88(0.70-1.10)	1.03(0.82-1.30)	0.69(0.54-0.89)	0.75(0.58-0.97)
*P*	0.008	0.273	0.264	0.764	0.005	0.030

Abbreviations: OS: overall survival; DMFS: distant metastasis-free survival; HR: hazard ratio; CI: confidence interval; ANPC: advanced nasopharyngeal carcinoma

We performed multivariate analysis to investigate whether leucopenia could be a marker of improved OS and DMFS (Table 3). Leucopenia and other prognostic factors, i.e., age, sex, T classification, N classification, pathological type, type of chemotherapy, paclitaxel use, and type of RT were included in the multivariate analysis, which determined that leucopenia, sex, T classification, and N classification were independent prognostic factors for OS and DMFS. Compared to patients without leucopenia, the hazard ratios (HRs) of death for patients with mild and severe leucopenia were 0.69 [95 % confidence interval (95 %CI) 0.56-0.85, *p* < 0.001] and 0.75 (95 %CI 0.59-0.95, *p* = 0.019), respectively. The HR of distant metastasis for patients with mild and severe leucopenia were 0.77 (95 %CI 0.61-0.96, *p* = 0.023) and 0.99 (95 %CI 0.77-1.29, *p* = 0.995), respectively. When we compared patients with mild leucopenia to patients with severe leucopenia, the HRs of death and distant metastasis were 0.93 (95 %CI 0.77-1.11, *p* = 0.416) and 0.77 (95%CI 0.64-0.93, *p* = 0.006), respectively.

**Table 3:** Multivariate analysis of survival for patients with ANPC

	All population		Cycles <4 population		Cycles > =4 population	
Variable	OS	DMFS	OS	DMFS	OS	DMFS
Leucopenia						
Mild VS Absent						
HR(95 %CI)^a^	0.69(0.56-0.85)	0.77(0.61-0.96)	0.70(0.55-0.89)	0.88(0.63-1.23)	0.73(0.46-1.15)	0.61(0.40-0.94)
*P*	<0.001	0.023	0.003	0.452	0.174	0.025
Severe VS Absent						
HR(95 %CI)^a^	0.75(0.59-0.95)	0.99(0.77-1.29)	0.82(0.60-1.11)	0.79(0.61-1.03)	0.97(0.74-1.27)	0.84(0.53-1.32)
*P*	0.019	0.995	0.204	0.083	0.828	0.446
Mild VS Severe						
HR(95 %CI)^a^	0.93(0.77-1.11)	0.77(0.64-0.93)	0.85(0.66-1.09)	0.90(0.68-1.19	0.71(0.46-1.07	0.73(0.56-0.96)
*P*	0.416	0.006	0.204	0.469	0.108	0.026
Gender						
HR(95 %CI)	0.67(0.55-0.81)	0.70(0.58-0.86)	0.66(0.52-0.84)	0.73(0.57-0.93)	0.66(0.48-0.91)	0.68(0.49-0.94)
*P*	<0.001	<0.001	0.001	0.013	0.010	0.019
Age						
HR(95 %CI)	1.82(1.57-2.12)	1.05(0.89-1.23)	1.88(1.55-2.28)	1.09(0.89-1.34	1.71(1.34-2.17)	1.03(0.81-1.33)
*P*	<0.001	0.532	<0.001	0.367	<0.001	0.783
T-classification						
HR(95 %CI)	1.49(1.35-1.66)	1.33(1.19-1.47)	1.51(1.33-1.72)	1.36(1.19-1.56)	1.49(1.26-1.76)	1.27(1.08-1.51)
*P*	<0.001	<0.001	<0.001	<0.001	<0.001	0.005
N-classification						
HR(95CI)	1.77(1.62-1.93)	1.78(1.62-1.97)	1.92(1.71-2.16)	1.92(1.68-2.18)	1.56(1.35-1.80)	1.63(1.40-1.90)
*P*	<0.001	<0.001	<0.001	<0.001	<0.001	<0.001

Abbreviations: OS: overall survival; DMFS: distant metastasis-free survival; HR: hazard ratio; CI: confidence interval; ANPC: advanced nasopharyngeal carcinoma

^a^Adjusted for age (<45 and ≥45 years old), sex, T classification (T1/T2/T3/T4), N classification (N0/N1/N2/N3), pathological type, type of radiotherapy, type of chemotherapy, and paclitaxel use

When pretreatment leukocyte count (≤10 × 10^9^/L vs. >10 × 10^9^/L) was included in the Cox model, leucopenia remained significant for OS (mild leucopenia: HR = 0.70, 95 %CI 0.57-0.86, *p* = 0.001; severe leucopenia: HR = 0.76, 95 %CI 0.59-0.97, *p* = 0.026) and DMFS (mild leucopenia: HR = 0.77, 95 %CI 0.61-0.96, *p* = 0.023; severe leucopenia: HR = 0.99, 95 %CI 0.77-1.30, *p* = 0.995).

Tables 2 and 3 depict the subgroup analysis results for patients who received <4 and ≥4 chemotherapy cycles. Mild and severe leucopenia tended to be associated with improved survival in patients who received <4 or ≥4 chemotherapy cycles.

## Discussion

In this study, we found that survival was improved in patients who developed leucopenia during chemoradiotherapy for ANPC. Patients with mild leucopenia had better OS and DMFS than those with severe leucopenia. Leucopenia was an independent prognostic factor for OS and DMFS in patients who received <4 and ≥4 chemotherapy cycles. This is the first instance that has been reported in pretreated ANPC.

As far as we know, leucopenia or neutropenia indicates that the chemotherapeutic agent dose is sufficient to cause bone marrow suppression and an anti-tumor effect [8, 9]. The absence of leucopenia or neutropenia indicates an absent or weak biological effect of chemotherapy, likely indicating that the dose is too low. On the other hand, severe leucopenia may indicate overdosage. High-dose chemotherapy does not improve survival, and impairs patient quality of life [28]. We speculate that moderate-dose chemotherapy, as evidenced by moderate toxicity, is the optimal treatment, correlating with better survival than under- or overdosage. Colleoni et al. [29] found that patients who received level II doses (65-84 % of the prescribed dose) had longer disease-free survival (DFS) and OS than patients who received higher (level I: >85 % of the prescribed dose) or lower (level III: <65 % of the prescribed dose) doses (*p* = 0.07, *p* = 0.03, respectively). Additionally, Brunetto et al. reported that there was no difference in OS for patients whose dose had been reduced compared to patients whose dose had been maintained [30]. Nakatat al. [17] and Shitara et al. [15] both found that patients with mild neutropenia had better outcomes than those with severe neutropenia; others have reported that patients who developed grade 2–3 leucopenia or neutropenia had significantly better prognosis than those with grade 4 leucopenia or neutropenia [16, 17, 31]. Our results agree with these results. In other words, mild leucopenia or neutropenia might be a barometer of the appropriate chemotherapeutic dosage to obtain sufficient anti-tumor effect in a patient, leading to improved clinical outcome; however, severe leucopenia or neutropenia might be a marker of overdosage and suboptimal survival.

However, there are differing findings: Kim et al. [22] reported that neutropenia was not a significant prognostic indicator of improved progression-free survival and OS (*p* = 0.180, *p* = 0.698, respectively) in stage I-IIIB breast cancer. Kumpulainen et al. [23] drew a wholly different conclusion, where the 10-year DFS in FIGO (International Federation of Obstetrics and Gynecology) stage IC-IV disease was 45 % in patients with lower leukocyte counts (<2.5 × 10^9^/L) and 66 % in patients with higher leukocyte counts (≥2.5 × 10^9^/L) (*p* < 0.05). The probable reason is that the different disease stages might obscure the impact of leucopenia. Most studies and ours studied patients with advanced-stage disease.

Several reports have stated that pretreatment high leukocyte or neutrophil count might be a poor prognostic indicator and that leucopenia or neutropenia are less likely to occur during treatment [32, 33]. However, in our multivariate analysis, which included this factor, leucopenia remained significant for OS and DMFS.

Due to the retrospective nature of our study, there are some limitations. First, the chemotherapy regimens and dose varied. Second, patients were identified from 2005 to 2010, and the normal range of hematological profiles may have varied. Third, although G-CSF was not used for prophylaxis, it would nevertheless affect the severity of leucopenia. Fourth, we only analyzed leucopenia, a sign of myelosuppression. Taking hemoglobin and platelet inhibition into account might reflect the relationship between myelosuppression and prognosis more accurately.

## Conclusions

Leucopenia during chemoradiotherapy of ANPC is strongly associated with better OS and DMFS; mild leucopenia indicates better survival than severe leucopenia. This may indicate that mild leucopenia is a surrogate marker for adequate chemotherapeutic dose. We can identify the patients who may benefit from chemotherapy if they experienced leucopenia during the treatment. The chemotherapy dose should not only depend on the body surface area, but also be based on its toxic effects. Prospective trials are required to assess whether dosing adjustments based on leucopenia may improve chemotherapy efficacy.

## Abbreviations

ANPC: Advanced nasopharyngeal carcinoma
CRT: Conventional radiotherapy
IMRT: Intensity modulated radiation therapy
NCCN: National comprehensive cancer network
G-CSF: Granulocyte colony-stimulating factor
MRI: Magnetic resonance imaging
ECT: Emission computed tomography
CT: Computed tomography
IC: Induction chemotherapy
CC: Concurrent chemotherapy
AC: Adjuvant chemotherapy
HR: Hazard ratio
CI: Confidence interval
OS: Overall survival
DMFS: Distant metastasis-free survival
LRFS: Locoregional relapse–free survival
DFS: Disease-free survival
FIGO: International federation of obstetrics and gynecology
WHO: World health organization
